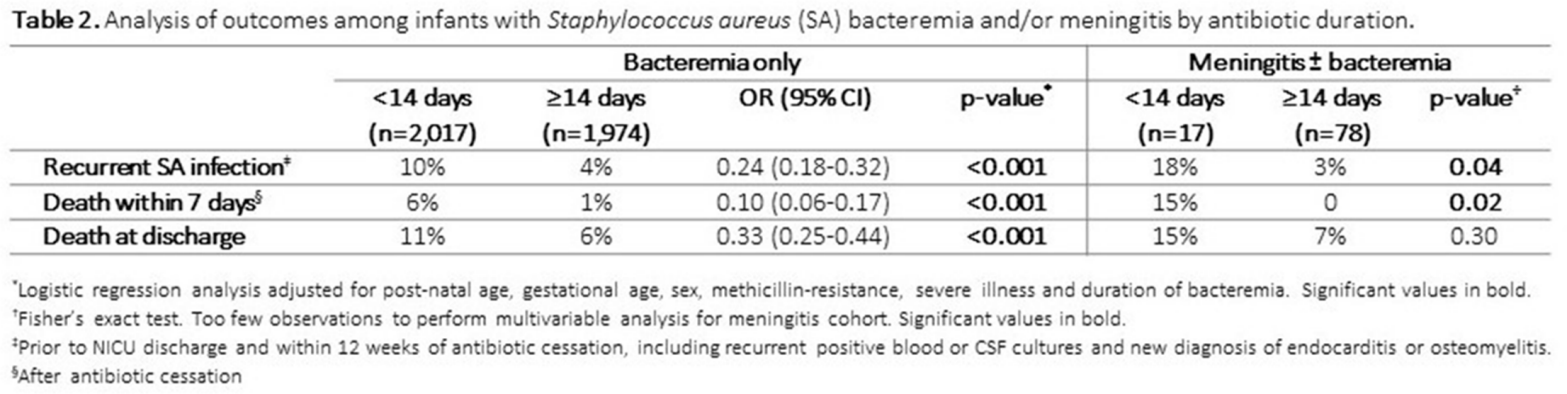# Association of Antibiotic Duration and Outcomes among NICU Infants with Invasive *Staphylococcus aureus* Infections

**DOI:** 10.1017/ash.2021.50

**Published:** 2021-07-29

**Authors:** Areej Bukhari, Ibukunoluwa Akinboyo, Kanecia Zimmerman, Danny Benjamin, Veeral Tolia, Rachel Greenberg

## Abstract

**Background:** *Staphylococcus aureus* is the second-leading cause of late-onset sepsis among infants in US neonatal intensive care units (NICUs). Management of *S. aureus* bacteremia and meningitis in infants varies widely due to the lack of standardized guidelines. We examined the association between initial antibiotic duration and recurrent *S. aureus* infection or death among NICU infants with *S. aureus* bacteremia and/or meningitis. **Methods:** We conducted a retrospective cohort study of infants in Pediatrix Medical Group NICUs from 1997 to 2018 with first episode of *S. aureus* bacteremia and/or meningitis, identified as having at least 1 blood or cerebrospinal fluid (CSF) culture growing only *S. aureus* at any point during their NICU stay. Excluded infants were those not started on antistaphylococcal therapy within 2 days of positive culture, those with had endocarditis or osteomyelitis, or those who died or were discharged during or up to 1 day after antibiotic cessation. Antibiotic cessation was defined as last day of antibiotic given if followed by at least 3 days without antibiotics. Multivariable logistic regression was used to analyze the association between antibiotic duration categorized as <14 or ≥14 days) and recurrent SA infection (within 12 weeks of antibiotic cessation, prior to hospital discharge), or death (within 7 days of antibiotic cessation and at discharge). **Results:** Of 4,086 infants included, 3,991 (98%) had *S. aureus* bacteremia only and 95 (2%) had meningitis ± bacteremia. Of those with bacteremia only, 2,017 (50.5%), and 17 (18%) of those with meningitis received <14 days antibiotics (Figure 1). Longer antibiotic duration was associated with lower gestational age, methicillin-resistance, severe illness and bacteremia duration of ≥4 days (Table 1). There was a significant association between <14 days antibiotics and recurrent infection (p = 0.04) and 7-day mortality (p = 0.02) in the meningitis cohort. Infants with SA bacteremia who received ≥14 days antibiotics had reduced odds of recurrent SA infection (OR 0.24, 95% CI 0.18-0.32) and death (OR 0.33, 95% CI 0.25-0.44), adjusting for post-natal age, gestational age, sex, methicillin-resistance, severe illness and duration of bacteremia (Table 2). **Conclusions:** In the largest study thus far examining antibiotic duration among hospitalized infants with *S. aureus* bacteremia and/or meningitis, ≥14 days antibiotics was associated with decreased odds of recurrent infection or death. Further studies are needed to define the optimal treatment duration and identify clinical factors distinguishing infants able to safely receive a shorter antibiotic duration.

**Funding:** No

**Disclosures:** None

**Figure 1.**
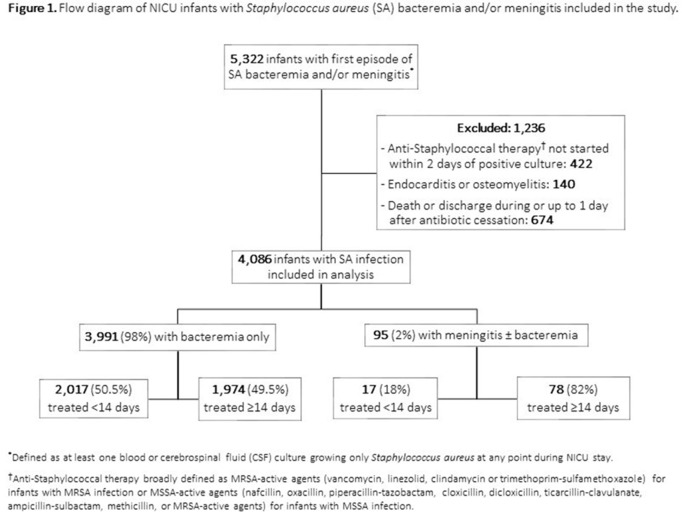

**Table 1.**
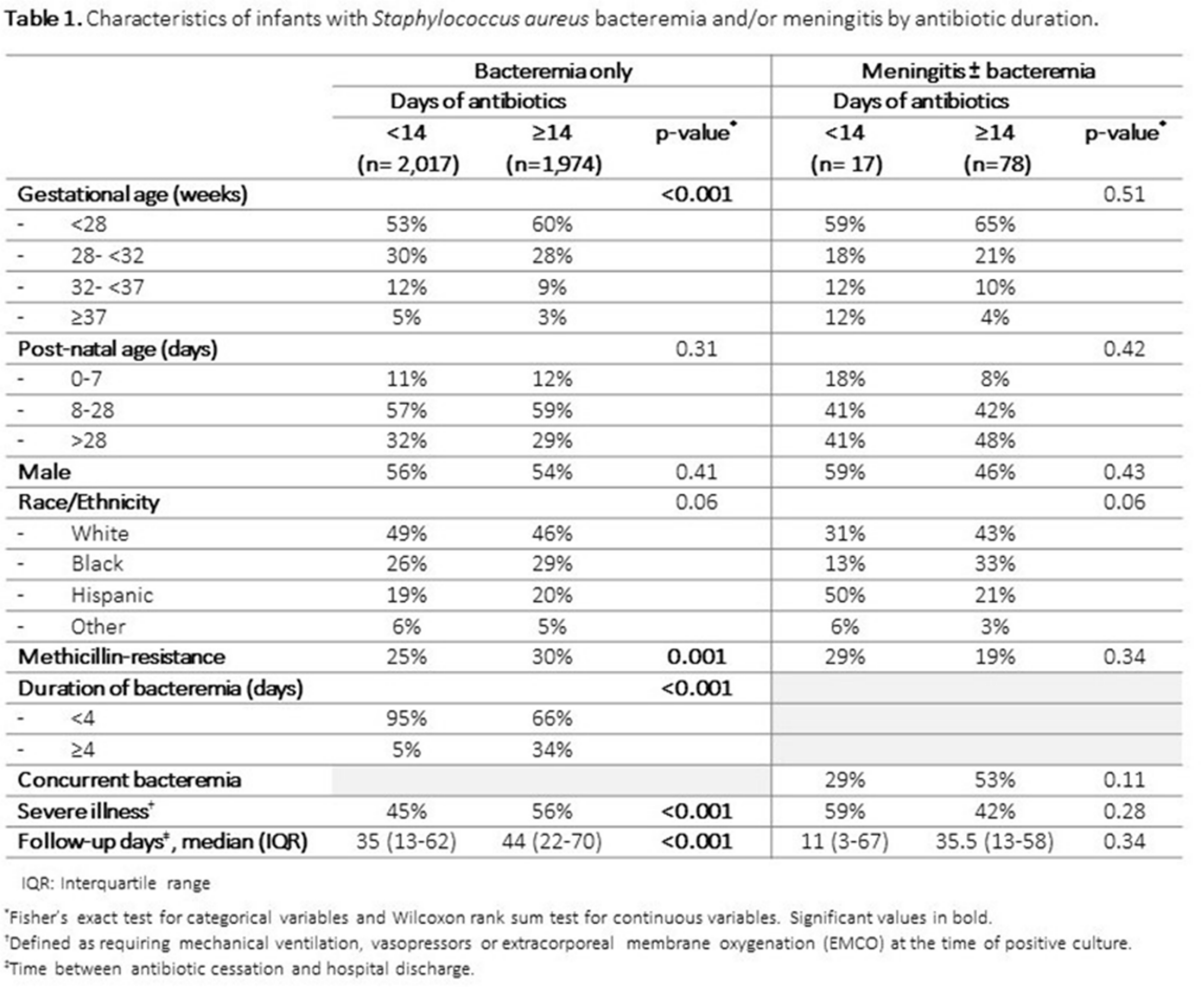

**Table 2.**